# Computer Aided Breast Cancer Detection Using Ensembling of Texture and Statistical Image Features

**DOI:** 10.3390/s21113628

**Published:** 2021-05-23

**Authors:** Soumya Deep Roy, Soham Das, Devroop Kar, Friedhelm Schwenker, Ram Sarkar

**Affiliations:** 1 Department of Metallurgical and Material Engineering, Jadavpur University, Kolkata 700032, India; sdrjumme@gmail.com (S.D.R.); sohamju22@gmail.com (S.D.); 2 Department of Computer Science and Engineering, Jadavpur University, Kolkata 700032, India; kardevroop@gmail.com (D.K.); raamsarkar@gmail.com (R.S.); 3 Institute of Neural Information Processing, Ulm University, 89081 Ulm, Germany

**Keywords:** breast cancer, IDC, machine learning, ensemble learning, feature selection

## Abstract

Breast cancer, like most forms of cancer, is a fatal disease that claims more than half a million lives every year. In 2020, breast cancer overtook lung cancer as the most commonly diagnosed form of cancer. Though extremely deadly, the survival rate and longevity increase substantially with early detection and diagnosis. The treatment protocol also varies with the stage of breast cancer. Diagnosis is typically done using histopathological slides from which it is possible to determine whether the tissue is in the Ductal Carcinoma In Situ (DCIS) stage, in which the cancerous cells have not spread into the encompassing breast tissue, or in the Invasive Ductal Carcinoma (IDC) stage, wherein the cells have penetrated into the neighboring tissues. IDC detection is extremely time-consuming and challenging for physicians. Hence, this can be modeled as an image classification task where pattern recognition and machine learning can be used to aid doctors and medical practitioners in making such crucial decisions. In the present paper, we use an IDC Breast Cancer dataset that contains 277,524 images (with 78,786 IDC positive images and 198,738 IDC negative images) to classify the images into IDC(+) and IDC(-). To that end, we use feature extractors, including textural features, such as SIFT, SURF and ORB, and statistical features, such as Haralick texture features. These features are then combined to yield a dataset of 782 features. These features are ensembled by stacking using various Machine Learning classifiers, such as Random Forest, Extra Trees, XGBoost, AdaBoost, CatBoost and Multi Layer Perceptron followed by feature selection using Pearson Correlation Coefficient to yield a dataset with four features that are then used for classification. From our experimental results, we found that CatBoost yielded the highest accuracy (92.55%), which is at par with other state-of-the-art results—most of which employ Deep Learning architectures. The source code is available in the GitHub repository.

## 1. Introduction

With the widespread digitization of health records, computer aided disease detection (CADD) systems that employ data mining and Machine Learning (ML) techniques have become increasingly commonplace. Considering the monstrosity of the disease, it comes as little surprise that the earliest efforts in CADD [1] started with mammography for the detection of breast cancer and were later extended to other types of cancer as well. Breast cancer is a condition in which the cells in the breast proliferate uncontrollably.

According to global cancer statistics [2], breast cancer in women has overtaken lung cancer as the most diagnosed cancer in the world, comprising almost 12% of the total instances of cancer worldwide. As is the case in most types of cancer, the treatment of breast cancer is greatly aided by early detection but involves surgical or intensive medical procedures if not diagnosed early.

Since treatment depends on the stage of cancer, one of the preliminary tasks of any pathologist involves a visual analysis of a histopathological slide stained with hematoxylin and eosin (H&E). It is possible to determine whether the tissue is in the Ductal Carcinoma In Situ (DCIS) stage or in the Invasive Ductal Carcinoma (IDC) stage from the slide. DCIS is a non-invasive cancer where cancerous cells have been found in the lining of the breast milk duct but have not spread into the surrounding breast tissue. However, in IDC, the abnormal cancer cells that began forming in the milk ducts spread beyond the ducts into other parts of the breast tissue. IDC is the most prevalent breast cancer type, responsible for about 70–80% of all breast cancer diagnoses. While being an important part of the process of treatment, IDC detection is an exceedingly time consuming and difficult task.

In light of the above situation, the problem of classifying histopathological slides into IDC(+) and IDC(-) has been delegated to ML using image processing techniques. ML relies on the extraction of feature descriptors from the image followed by classification. These descriptors include color descriptors, like RGB histograms; statistical descriptors, like Haralick features; and texture descriptors, like Speeded-Up Robust Features (SURF), etc.

Though significant research has been devoted to classification using either of the feature descriptors, very little has been done to agglomerate the information furnished by each descriptor. The alternative to ML is the usage of neural networks in DL, which dispenses with the need for hand-crafted features. However, this comes at the cost of greater complexity and the need for higher computational power. In addition, to train a DL model properly, there is a requirement of a huge amount of input samples, which sometimes may not be available for real-life problems.

Keeping the above facts in mind, in this paper, we attempted to classify the histopathological images into IDC(+) and IDC(-) using an ensemble of texture and statistical features. The accuracy of texture features, such as Scale Invariant Feature Transform (SIFT) [3], SURF [4], Oriented FAST and rotated BRIEF (ORB) [5] and Gray Level Co-Occurrence Matrix (GLCM), as well as statistical features, such as Haralick features [6] are computed using various standard ML algorithms, such as Random Forest (RF) [7], CatBoost (CB) [8] and AdaBoost (AB) [9]. Such scores are then compared with the accuracy metrics for various combinations of these features. The best results are obtained by ensembling all features using a stacking method. However, as explained later, the stacking ensemble has been tweaked to weed out redundant features using Pearson’s Correlation Coefficient based feature selection.

Keeping the above facts in mind, in this work, we made an exhaustive set of experiments as well as comparisons using the well-known statistical and texture descriptors to identify the best subset of features, which contribute to differentiate between cancerous and non-cancerous cells. We also identified a set of ML classifiers that learn complimentary features, which were aggregated to provide a better feature set. While an ensemble approach is well-known in the literature; here, we have been able to find the correct blend of statistical and texture descriptors to achieve better results, and we have been able to even reduce the dimension of the feature vector without compromising the classification accuracy.

**Figure 1:**
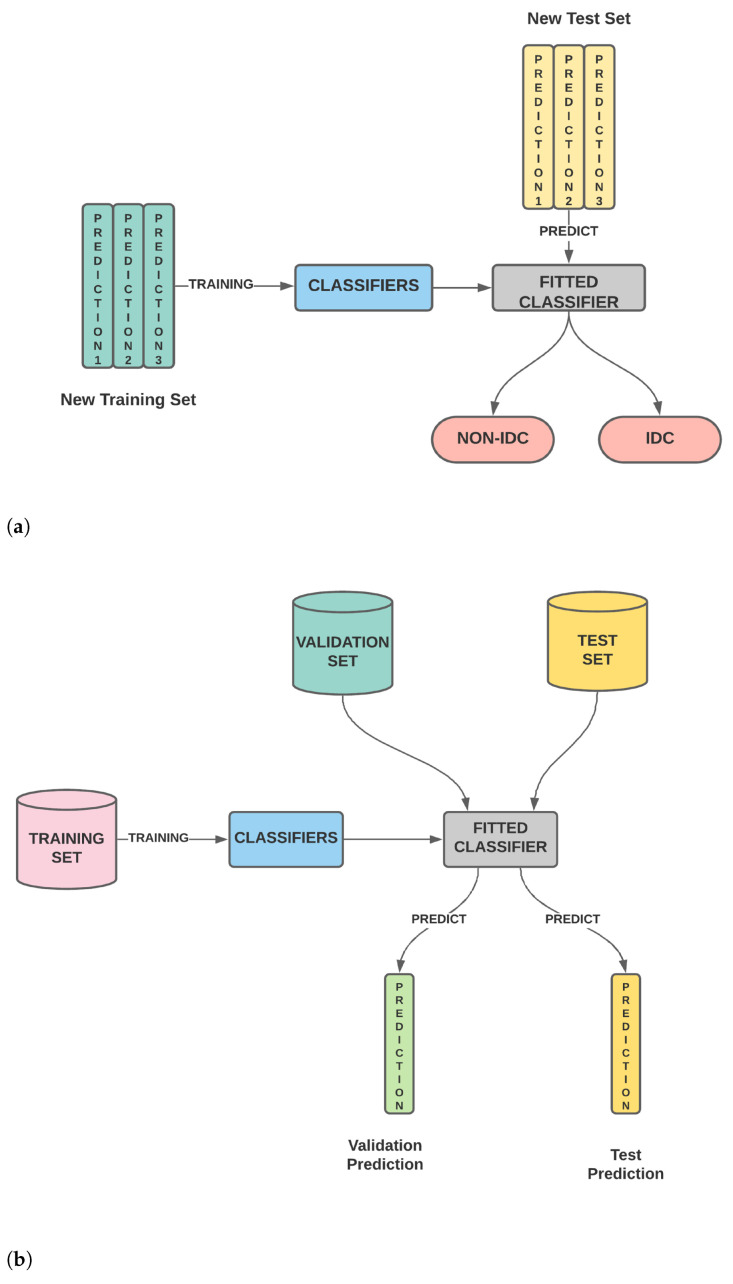

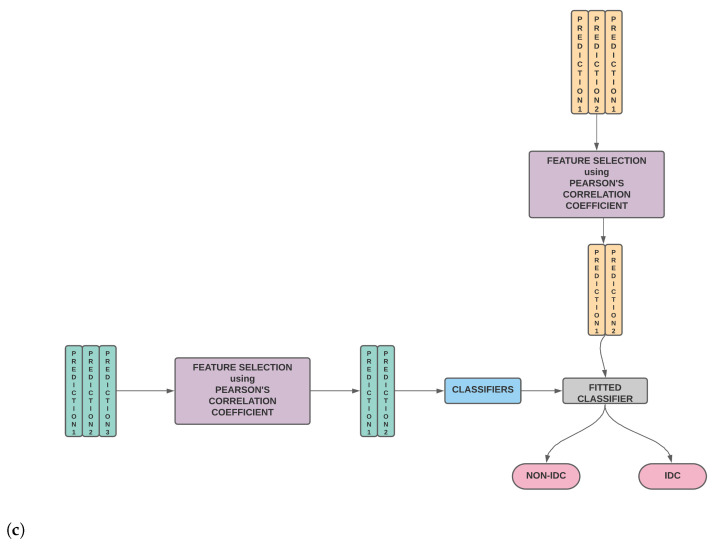
(**a**) The classifier is trained on the training set. The trained classifier is then used to predict the outcome of the validation set as well as the test set. (**b**) The second stage wherein the validation predictions for different classifiers are stacked to generate the new training features while the test predictions for different classifiers are stacked to generate the new test features. Using these new training and test features, the classifier is evaluated. (**c**) Our model wherein the second stage is modified and we perform feature selection on the new training and test features before model training and testing.

In a nutshell, the contributions of our proposed work are as follows:

1. An ensemble method of texture and statistical descriptors followed by classification using ML classifiers, like RF and CB, is proposed. The approach used for ensembling is stacking.
2. The conventional method of stacking is modified to weed out potentially redundant features using Pearson’s Correlation Coefficient based feature selection—the details of which are elaborated in Figure 1.
3. A thorough comparison enumerating the relative performances of SURF, SIFT, ORB, GLCM and Haralick and various combinations of the same is presented.
4. A light-weight ML model is created, which is computationally inexpensive and algorithmically easy to design. It is equally, if not more, accurate than other existing methods.
5. The experimental results demonstrate the superiority of the proposed model over the standalone feature descriptors.

The remaining paper is arranged as follows: Section 2 details the research endeavors pertaining to the IDC(+) predictions found in the recent literature. Section 3 provides our proposed model along with the relevant dataset description, while the experimental results are enumerated and discussed in Section 4. Finally, the paper concludes in Section 5 along with some future research directions.

## 2. Literature Survey

Over the last few years, researchers have suggested various methods for breast cancer diagnosis in histopathology images. New innovative methodologies are being proposed that include traditional machine learning and deep learning architectures. Many research works have placed emphasis on feature extraction. Textural and statistical features are often used. Doyle et al. [10] made use of 64 Gabor filter features, 15 statistical gray-level features, graph-based features, 16 second-order statistical features and 24 nuclear features per each of three color channels (hue, saturation and intensity) the in HSI color space utilizing three distinct window sizes.

Dundar et al. [11] created an automatic classification system for classifying breast microscopic tissues into actionable subtypes (atypical ductal hyperplasia and ductal carcinoma in situ) and usual ductal hyperplasia. Statistical features, like the perimeter, mean gray-level intensity and the ratio of major to minor axis of the best-fitting ellipse, were utilized to model the cell size, shape and nucleoli appearance, respectively (histological descriptors). Niwas et al. [12] proposed a method where they extracted first-order statistical and second-order statistical features.

With the help of the Log–Gabor complex wavelet bases, the color texture features of the segmented nucleus were assessed. The aforementioned techniques include integrating various forms of intricately handcrafted features to reflect the visual content of breast cancer histopathological images. In this context, extracting 59 image features using a Local Binary Pattern (LBP) based feature descriptor for breast mammography yielded an accuracy of 84% on an Support Vector Machine (SVM) classifier with a polynomial kernel [13]. Even statistical features were investigated in computer aided breast cancer detection by Yasiran et al. [14].

Narayanan et al. [15] came up with a novel deep CNN architecture. Images were downsized from 50×50 to 48×48. In the pre-processing stage, a color constancy technique and histogram equalization were applied. The pre-processed images were then fed into the CNN. The proposed architecture was made out of five convolutional layers followed by a fully connected (FC) layer and a softmax layer. Images that were pre-processed using color constancy techniques resulted in better performance metrics compared with images pre-processed utilizing Histogram Equalization.

Debelee et al. [16] extracted features using CNN and employed Principal Component Analysis for dimensionality reduction. k-Nearest Neighbors (k-NN) was utilized to classify mammograms as normal or abnormal. Debelee et al. [17] extracted features from images using pre-trained Inception-V3 and their proposed modified adaptive K-means (MAKM) method. They collated images from the local and public datasets. GLCM and Gabor (Texture features) from ROIs and CNN-based extracted features were fused and fed into five classifiers (SVM, k-NN, NB, MLP and RF) to quantify the descriptive power of the features.

Rahman et al. [18] propounded a multilayered CNN. The IDC dataset has a significant class imbalance. An equal number of images were picked out from both the IDC(+) and IDC(-) classes in order to eradicate the class imbalance problem. The selection of images was performed randomly to avoid bias. Overfitting was mitigated by applying various data augmentation, viz; random 10-degree rotation, shifting, zooming and flipping horizontally and vertically. The implemented architecture achieved an accuracy of 89%.

Romano and Hernandez [19] also rectified the class imbalance present in the IDC(+) dataset. The images were normalized, and data augmentation was carried out in order to prevent overfitting. The images were augmented utilizing an array of random transformations so that the classifier would never twice view the exact same image. The proposed CNN architecture consists of two convolutional layers, a new pooling layer, called accept–reject pooling, dropout layers and FC layers. After feeding the images into the CNN, the model yielded the best performance with an accuracy of 85.41%.

Cruz-Roa et al. [20] proposed a three-layer CNN architecture for the automatic detection of IDC tissue regions in whole slide images (WSI). Their CNN has 16, 32 and 128 neurons in the first and second convolutional-pooling layers and the FC layer, respectively. Wang et al. [21] extended the work of the Cruz-Roa et al. [20] architecture to four different architectures. They also concluded that data augmentation was not efficacious in the automatic detection of breast cancer with the IDC(+) dataset. Segmenting irregularities were expunged. There were a plethora of images that were not 50×50 and had to be removed. All the redundant images with 90% black space or 90% white space were removed.

Instead of giving equal significance to all the patches, the authors in [22] presented an attention mechanism that allowed the network to focus on the relevant features of patches. A weighted representation of all the constituent patches of an image was used for learning. Sanyal et al. [23] came up with a novel hybrid ensemble framework made of many fine-tuned CNNs, such as supervised feature extractors and XGBoost as a top level classifier. They used high resolution histopathology images for patch-wise detection.

Recently, Chapala and Sujatha [24] utilized pre-trained ResNet-50 and ResNet-34 mechanisms. The dataset was split into three different ratios of training and testing datasets, such as 90%–10%, 75%–25% and 50%–50%. ResNet-50 outperformed ResNet-34 in terms of accuracy. In [25], Debelee et al. presented a detailed review of DL techniques used in breast cancer image analysis. They reviewed recent publicly available databases of breast cancer images and discussed the modalities that are used for breast cancer imaging.

## 3. Proposed Method

**Figure 2:**
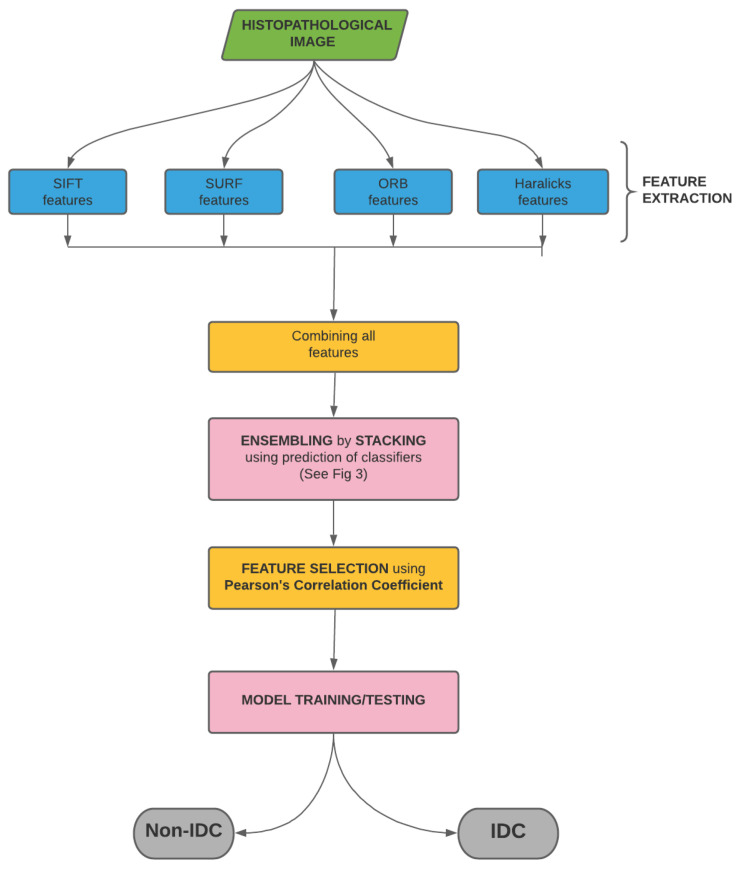
Pipeline of the proposed model used for breast cancer detection from histology images. We start with the histopathological image from which we extract 256 SIFT, 256 SURF, 256 ORB and 14 Haralicks features. The 782 (= 256 + 256 + 256 + 14) features are then combined. These features are then ensembled by stacking. In order to weed out redundant features, we use Pearson’s Correlation Coefficient. This is followed by model training and testing, which eventually classifies the images as IDC(-) and IDC(+).

In the present work, we classify IDC(+) and IDC(-) images by extracting their textural features, like SIFT, SURF and ORB, and their statistical features, like Haralick features. After extraction, these features are combined and then ensembled by stacking. The stacked features are then used to obtain the final prediction. The pipeline of the entire process is shown in Figure 2. In the succeeding sections, we discuss the data, the extracted features and the process of stacking in detail.

### 3.1. Dataset Description

**Figure 3:**
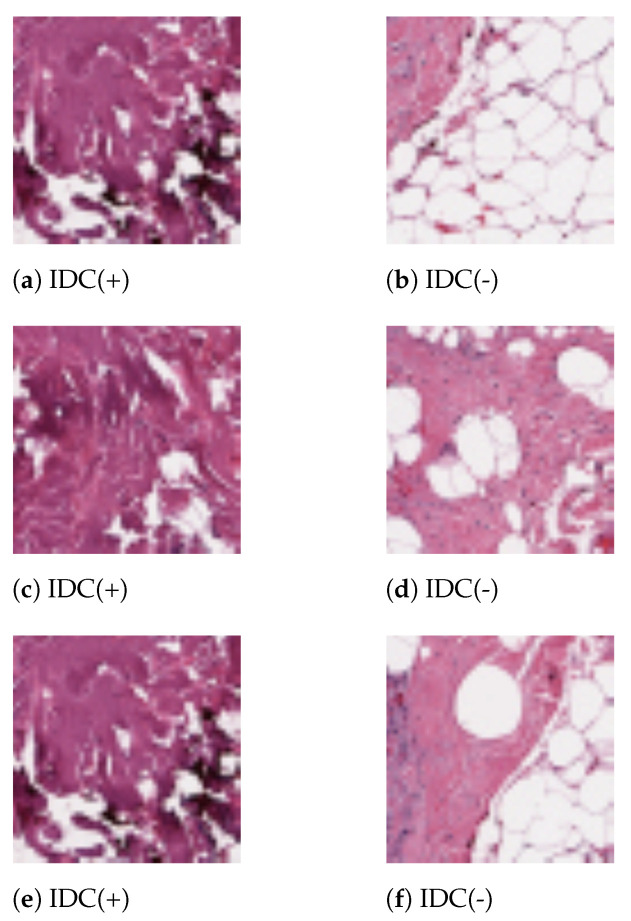
Sample images of the present dataset. (**a**,**c**,**e**) IDC(+) patches. (**b**,**d**,**f**) IDC(-) patches.

In this work, we have utilized the IDC Breast Histopathology Image Dataset. The original dataset consists of 162 WSI of breast cancer specimens scanned at 40×. 277,524 patches of each resolution 50×50 were extracted from those WSI. Out of this, 198,738 images were IDC(-) and 78,786 were IDC(+). Sample histopathology images for both the categories from the present dataset are shown in Figure 3. The dataset is publicly available in the link https://www.kaggle.com/paultimothymooney/breast-histopathology-images (accessed on 17 December 2020).

### 3.2. Feature Extraction

In order to identify the relevant features that will differentiate the IDC(+) and IDC(-) image patches, both texture-descriptor-based features and the statistical features of the images were considered. Texture descriptors, like SIFT, SURF and ORB, as well as statistical features, such as Haralick features, which are based on GLCM, were used. Each texture descriptor, i.e., SIFT, SURF and ORB, revealed 256 features, while the Haralick comprised 14 features. These features were appended to create a dataframe that is used in the subsequent processes. The feature descriptors used to create the dataframe are described here.

#### 3.2.1. Scale-Invariant Feature Transform

SIFT is a feature extraction method used to detect and describe local features in images. In 2004, D. Lowe [3] proposed this algorithm, which extracts keypoints to calculate its descriptors. SIFT is invariant to the image scale as well as rotation. SIFT object keypoints are first retrieved and stored in a database from a collection of reference images. In a new image, an object is recognized by analogizing each attribute from the new image to a database. We then look for candidates to complement the feature vectors dependent upon the Euclidean distance. Each feature in this image is compared with the database and corresponding candidate features from its Euclidean distance based on feature vectors in order to identify the object in a new image.

To determine consistent clusters efficiently, a hash table implementation of a generalized Hough transform is utilized. Each cluster of three or more features that point to the presence of an object and orientation are then subjected to an additional thorough model verification, and subsequent outliers are discarded. Finally, given the accuracy of the fit and the number of possible false matches, the likelihood that a certain set of features indicates the presence of an object is computed. Object matches that pass all of these tests are considered correct.

#### 3.2.2. Speeded-Up Robust Features

In computer vision, SURF is a local feature detector and descriptor used for object recognition, image registration, classification etc. Though inspired by the SIFT descriptor, SURF performs much faster than SIFT. The SURF feature detector functions by applying an approximate Gaussian second derivative mask to an image at different scales. It is more robust to rotation when compared to the Harris corner method. The method is extremely fast because it uses an integral image. Here the value of a pixel (x,y) is the sum of all values in the rectangle defined by the origin and (x,y). In such an image, the sum of the pixels within a rectangle of any size in a source image can be found by four operations. This allows a rectangular mask of any size to be applied with very little computing time. SURF utilizes wavelet responses in the horizontal and vertical directions to allocate the orientation, which can be found using integral images.

#### 3.2.3. Oriented FAST and rotated BRIEF

With an intention of providing a efficient and fast alternative to SIFT, Rublee et al. [5] devised a fast robust feature detector, ORB. A FAST keypoint detector and a modified version of the visual descriptor BRIEF (Binary Robust Independent Elementary Features) are the cornerstone of ORB.

ORB performs as well as SIFT on the task of feature detection while being a hundred-times faster. Both the underlying detectors of ORB, i.e., the FAST keypoint detector and the BRIEF descriptor, provide good performance at a low cost. The main contributions of ORB are as follows:

- The addition of an orientation component to FAST.
- The efficient computation of oriented BRIEF features.
- A learning method for decorrelating BRIEF features under rotational invariance leading to better performance in nearest-neighbor applications.
- Analysis of variance and correlation of the oriented BRIEF features.

#### 3.2.4. Haralick Features

Haralick texture features are calculated based on a Gray Level Co-occurrence Matrix, (GLCM). A GLCM is a matrix that counts the distribution of gray levels between neighboring pixels in the image. Since co-occurrence matrices are typically large and sparse, different metrics of the matrix are frequently used to obtain a more relecant set of features. Robert Haralick described 14 statistical features that can be determined from the co-occurrence matrix in order to describe the texture of the image:

- Correlation
- Variance
- Inverse Difference Moment
- Sum Average
- Sum Variance
- Sum Entropy
- Entropy
- Information Measure of Correlation 2
- Maximum Correlation Coefficient
- Difference Variance
- Difference Entropy
- Information Measure of Correlation 1
- Angular Second Moment
- Contrast

The process of extracting the above mentioned features is outlined in the following Algorithm 1.

### 3.3. Ensembling by Stacking

Ensemble learning is an ML paradigm where multiple models, colloquially known as “weak learners”, are trained to solve the same problem and combined to obtain better results [26]. The main idea is that, when weak learners are correctly combined, we can obtain more accurate and robust models [27]. Ensemble methods include stacking, blending, boosting and bagging [28] and have been applied successfully in various regression and classification tasks [29] as well as in clustering [30] and reinforcement learning [31].

Stacking is the process of learning multiple weak learners and combining them by training a meta-model to produce predictions based on the multiple predictions returned by these weak models. Stacking differs from other ensemble algorithms in that it considers heterogeneous weak learners in the form of different types of algorithms.

For our classification task, the algorithms chosen as weak learners include XGBoost (XGB), AB, CB, RF, Extra Trees (ET) and Multi Layer Perceptron (MLP). The fused dataframes containing SURF, SIFT, ORB and Haralick features are used for stacking. These are comprised of the following steps:

1. Split the training data in two folds.
2. Fit the learners, i.e., XGB, AB, CB, RF, ET and MLP, in our use case to the first fold.
3. For each of the learners, make predictions for the validation data and test data.
4. Using the predictions on the validation set and test set as new training and test features, training and test the aforementioned classifiers.

Thus, stacking is, in essence, a combination of two models, the base model, which fits on the training data and whose predictions are compiled, and the meta model, which learns how to best combine the predictions generated by the base model. In practice, after step 3, we check the Pearson’s Correlation Coefficient among the newly created features to weed out potentially redundant features and then make the final predictions. The process of stacking along with our modifications is shown in Figure 1.

Hence, we start with 782 features after combining the SIFT, SURF, ORB and Haralick features. After step 2, we end up with six features corresponding to the predictions of the six classifiers. These six features may contain overlapping redundant information that needs to be filtered. After feature selection using the Pearson Correlation Coefficient, we are left with four features. These four features are then trained and tested using various classifiers. Out of all the classifiers, CB yielded the highest accuracy (92.55%) after stacking. The details of the learners used for stacking, i.e., RF, XGB, AB, CB, ET and MLP, are provided below.
**Algorithm 1:** Extract the SIFT, SURF, ORB and Haralick Features.
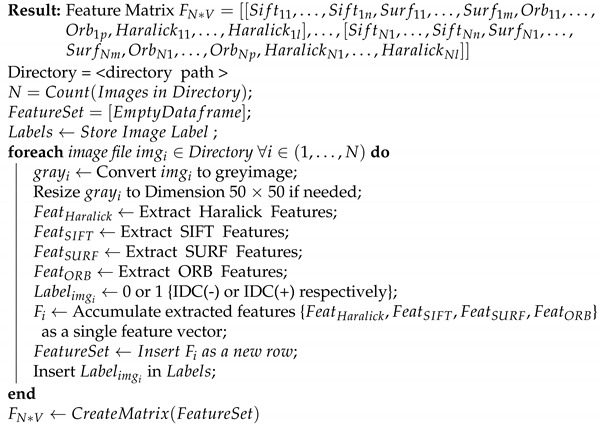


#### 3.3.1. Random Forests

Random Forests (RF) uses an ensemble of Decision Trees (DT) to predict classes. RF uses bootstrap aggregation or bagging of tree learners. Given a training set $X=\{x_{1},x_{2},\ldots ,x_{n}\}$ with labels $y=\{y_{1},y_{2},\ldots y_{n}\}$ bagging iteratively selects a random sample with replacement of the training set and fits trees to these samples, *B* times

For b=1,…,B:

1. We sample, with replacement, *n* training examples from *X* with corresponding labels from *Y* and name them $X_{b}$ and $Y_{b}$.
2. We train a classification tree on $X_{b}$ and $Y_{b}$.

To test the samples, RF classifies the samples by the majority voting of its decision trees. It outshines the simple decision tree because it does not rely on only one tree for classifying a test sample. Hence, it overcomes the problem of overfitting that is frequently seen in DTs.

#### 3.3.2. Extremely Randomized Trees

Extremely Randomized Trees, also known as Extra Trees, or ET [32] in short, is an ensemble ML algorithm. Algorithms, such as bootstrap aggregation (bagging) and random forest, are an ensemble of DTs. The algorithm creates multiple unpruned decision trees from a training dataset. In the case of regression, predictions are made by averaging the predictions of the decision trees. For classification, we resort to majority voting of the decision trees.

#### 3.3.3. Extreme Gradient Boosting

Extreme Gradient Boosting (XGB) [33] is a new algorithm that utilizes the gradient tree boosting concept. XGB was developed to improve the speed and performance of stack-based DT classifiers. XGB also introduced regularization parameters to address the problem of overfitting. Gradient boosted trees use regression trees sequentially as weak learners.

#### 3.3.4. AdaBoost

The AdaBoost (AB) algorithm, short for Adaptive Boosting, is a boosting technique that is used as an ensemble method in ML. It is called Adaptive Boosting as the weights are re-assigned to each instance, with higher weights to the incorrectly classified instances. Boosting is used to reduce bias as well as the variance for supervised learning. In AB, learners are grown sequentially. Except for the first, each learner is grown from previously grown learners. In this way, weak learners are converted into strong ones.

#### 3.3.5. CatBoost

CatBoost (CB) is an open-source ML algorithm from Yandex. The name “CatBoost” comes from merging two words—“Category” and “Boosting”. The algorithm works well with varied categories of data, like text, audio and images. CatBoost is based on a gradient boosting library. It allows us to operate with categorical features without any explicit pre-processing.

#### 3.3.6. Multilayer Perceptrons

A Multilayer Perceptron (MLP) is a feed-forward artificial neural network that generates a set of outputs from a set of inputs. An MLP is characterized by different layers of input nodes connected as a directed graph between the input and output layers. MLP uses backpropagation for training the network. For our use case, a six-layered network with 100, 50, 20 and 10 hidden layer neurons was used.

## 4. Experimentation and Analysis

This section describes the outcomes of the proposed stacking ensemble of the SURF, SIFT, ORB and Haralick features. Here, we discuss the performance metrics used to evaluate the performance of our proposed model using the said ML classifiers followed by a summary of the comparative performance among the said model, standalone feature descriptors and various combinations of feature descriptors. We also compared the performance of our model with some state-of-the-art (SOTA) methods found in the literature. We utilized the Google Colab CPU, Python 3.7 and OpenCV 3.4.2.17. The training, testing and validation procedures were executed in the Google Colab platform. The proposed model had an average runtime of 33 seconds for data prepossessing and training. Once trained, the model classified a test image in 117.62 milliseconds.

### 4.1. Results and Analysis

After pre-processing the images of the said dataset, we extracted texture descriptors, like SIFT, SURF and ORB, and statistical descriptors, like Haralicks. These features were subsequently stacked. This involved two stages: first, the descriptors were used as features to fit the base learners, like XGB, RF, AB, CB, ET and MLP. The learned models were then used to first predict on the training set and then on the test set. The predictions of the training and test sets for each of the base learners are ’stacked’ to generate new training and test sets.

**Figure 4:**
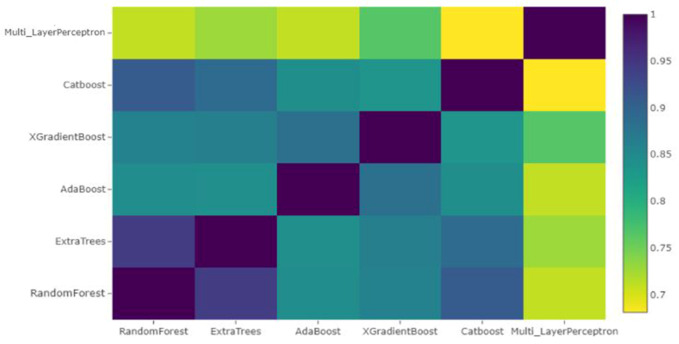
Pearson Correlation between features after stacking.

The new training and test sets now have only six columns corresponding to the predictions of the six learners. The Pearson correlation of these features is shown in Figure 4. The heatmap shows that the models MLP, XGBoost, Random Forest and AdaBoost were complementary according to the correlation between their predictions. Therefore, these models were selected to construct a stacked ensemble model. This was done in order to avoid the problem of multi-collinearity. The trained classifiers may convey the same information, and enhanced feature selection is essential to check redundancy of features. In the second stage, we used the new dataset containing only predictions of the aforementioned four algorithms to fit the different classifiers. The comparative performance of the classifiers is provided in Table 1.

**Figure 5:**
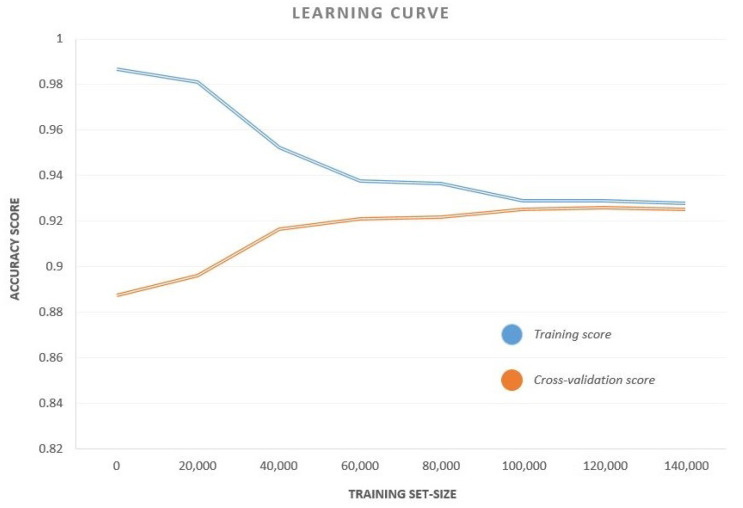
The Accuracy–Training Set Size Curve for the CB classifier. The curve plots the test accuracy for different training set sizes keeping the test set unaltered.

We observed that CB had the highest accuracy, recall and F1-Score among all the classifiers, while MLP has a perfect precision score. A curve of the accuracy versus training set size for CB classifier is also shown in Figure 5. The curve plots the test accuracy for various sizes of training sets keeping the test set unaltered. This curve helps us to analyze the variation of training and test accuracies with an increase in data samples. As is evident from the curve, both the training and cross-validation accuracies remained remarkably constant beyond a training set size of 50,000. This means that, even for a relatively small sample space, the results are reproducible.

Comparison of machine learning models using test accuracy is a rather shallow approach to zero in on an algorithm. When comparing two models, we want to ascertain that the differences between the scores are statistically significant and not due to chance or noise. We used the paired *t*-test using the 5 × 2 procedure, i.e., two-fold cross-validation with five repeats using a significance level of 5%. Table 2 shows the p-score for different pairs of models. We note that the CB classifier has a p-score lower than 0.05 for every classifier combination establishing, thereby, that the proposed superiority of the CB classifier is not due to a statistical fluke.

**Figure 6:**
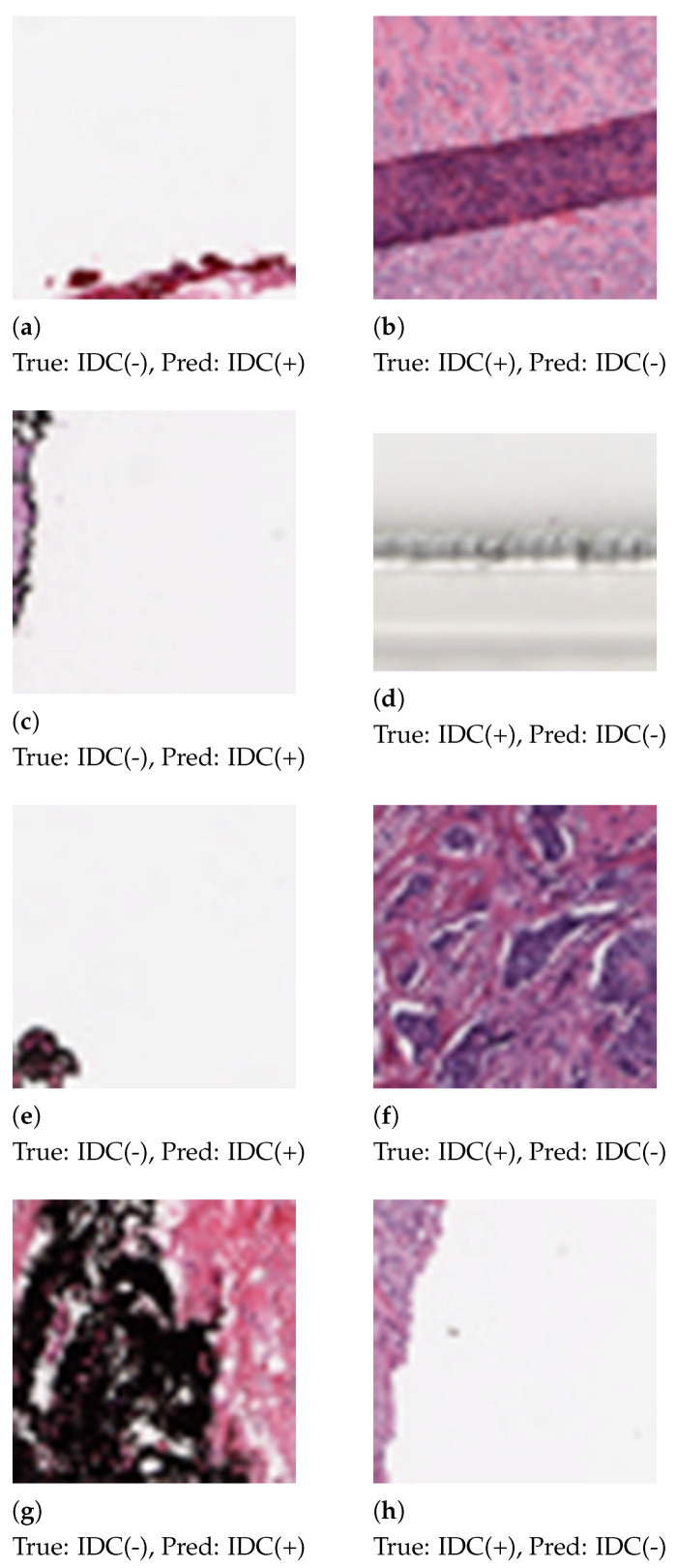
Patches that are misclassified by the proposed method. In the first column (**a**,**c**,**e**,**g**) are false positive errors. In the second column (**b**,**d**,**f**,**h**) are false negative errors.

Some patches, as shown in Figure 6, were misclassified by the proposed method. We analyzed the histograms of the misclassified images and observed the skewed distribution of color channels. Patches that contain 92%–99% white space or black space are heavily misclassified. Patch extraction from the Whole Slide Image (WSI) also introduced noise in the patches.

### 4.2. Comparison with Standalone Descriptors

To confirm the superiority of our model, we compared it with simple models. In the first leg of the experimentation, we used simple feature descriptors, like SIFT, SURF, ORB and Haralick, to train our model. The accuracy for various classifiers after training on these feature descriptors is described in Table 3. CB had the highest accuracy for SIFT, SURF and ORB while MLP had the highest accuracy for Haralick and GLCM. However, it is much lower than the accuracy obtained on stacking the features.

In the second leg of experimentation, we attempted various combinations of feature descriptors and used the same set of classifiers. The accuracy of the classifiers on these combined descriptors is reported in Table 4. Even in this case, CB had the highest accuracy for the SIFT-SURF, SIFT-ORB and ORB-SURF combinations, and XGB outperformed the SIFT-SURF-ORB combination. However, the highest accuracy of 0.87 for the SIFT-SURF combination was lower than the accuracy recorded for the stacked ensemble.

### 4.3. Comparison with Some SOTA Methods

In this section, we compare our proposed method with several SOTA methods that were proposed to detect breast cancer utilizing histopathological images. Table 5 presents the comparative results of various SOTA methods reported in the literature with their respective proposed method.

Alghodhaifi et al. [34] proposed IDCNet and IDCDNet. They implemented various activation functions, such as Sigmoid, ReLU and Tanh, to test the robustness of the models. The IDCNet model with ReLU activation outdid IDCDNet and yielded an accuracy of 87.13%. In [20], Cruz-Roa et al. proposed a three-layer CNN model with 16, 32 and 128 neurons for the first and second convolutional-pooling layers and the FC layer, respectively. The said model obtained a balanced accuracy (BAC) of 84%. Wang et al. [21] extended the architecture of [20] and created four different architectures.

Redundant images with inconsistent sizes were expelled. The comparative study made on the proposed CNNs reported that the five-layer, 50% dropout CNN architecture fetched an accuracy of 89% and a BAC of 87%. Transfer learning was employed by Chapala and Sujatha [24]. Pre-trained ResNet-50 and ResNet-34 were used for classification. ResNet-50 performed ResNet-34 in terms of accuracy with a 90:10 data split.

Some authors, such as Rahman et al. [18] and Romano and Hernandez [19], addressed the profound class imbalance present in the IDC(+) dataset. Rahman et al. [18] expunged the class imbalance issue by randomly selecting an equal number of images from both the IDC(+) and IDC(-) classes. They alleviated overfitting by applying various data augmentation techniques. The Xavier initialization method was used to initialize the weights of the convolution layers of the architecture. The proposed CNN achieved an accuracy of 89%. Romano and Hernandez [19] sorted out the class imbalance, normalized the images and carried out data augmentation. The proposed CNN consisted of two convolutional layers, accept–reject pooling layers, dropout layers and FC layers. The model attained an accuracy of 85.41%.

The methods discussed thus far utilize deep neural network architectures. CNN models proposed by authors, such as [18,19,20,21,24], use 100352, 3136, 9216, 30976 and 7200 features, respectively, for the classification of images into IDC(+) and IDC(-) classes. In comparison, our model uses a machine-learning-based approach and utilizes textual (SIFT, SURF and ORB) and statistical (Haralick) features followed by stacking.

We started with 782 features after combining the SIFT, SURF, ORB and Haralick features, which eventually reduced down to four features for decision making. However, this lowering of the number of features did not come at the cost of a decrease in performance. Our model achieved comparable results without using techniques, such as data augmentation and imbalance correction. This is in contrast to some of the models described above. In essence, our model achieved superior results with a more lightweight model.

## 5. Conclusions and Future Direction

Breast cancer is a deadly disease among women that kills more than half a million people every year. However, the chances of survival and longevity increase substantially with early detection. According to the American Cancer Society, the 5-year survival rate for detection in the localized stage is 99%, i.e., 99 out of 100 patients detected in the localized stage survive 5 years or beyond. On the other hand, the survival rate drops to 88% if the cancer is regional. Hence, early detection can go a long way in treatment, and image classification using ML and DL techniques can expedite this process.

In this paper, we observed the impact and contribution of a variety of image features. Both statistical features derived from the GLCM and image texture features, like SURF, SIFT and ORB, were investigated, and various combinations were used to produce the most important set of features to produce the highest accuracy. An ensemble of different classifier models was designed after observing the correlations in their individual predictive capacities. Finally, the CB classifier trained on the modified set of features achieved an accuracy of 92.55%. However, further improvements can still be made.

Different types of feature combinations can be used. Features extracted from DL models, like VGG, ResNet etc., can be combined with textural and statistical features to create more robust models. Feature selection based on optimization algorithms can also be employed to improve the predictive power. It is also noteworthy that the dataset is highly imbalanced. This can be rectified by using Generative Adversarial Networks (GANs) to create IDC(+) histopathology images.

## Figures and Tables

**Table 1:** The comparative performance of the classifiers used for the classification of IDC(+) and IDC(-) images after stacking.

MODEL	Accuracy	Precision	Recall	F1-Score
**XGB**	0.882	0.881	0.886	0.883
**MLP**	0.894	1.000	0.752	0.858
**CB**	0.922	0.931	0.887	0.908
**RF**	0.871	0.882	0.870	0.875
**AB**	0.879	0.883	0.881	0.882
**ET**	0.897	0.885	0.875	0.880

**Table 2:** P-scores for the paired *t*-test between classifiers. Each cell represents the p-score obtained after implementing the paired *t*-test between the corresponding row and column. The left diagonal has been left blank as it represents the paired *t*-test between a classifier and itself.

	XGB	RF	AB	CB	MLP	ET
**XGB**	-	0.048	0.043	0.023	0.053	0.048
**RF**	0.048	-	0.034	0.019	0.025	0.033
**AB**	0.043	0.034	-	0.036	0.043	0.032
**CB**	0.023	0.019	0.036	-	0.037	0.023
**MLP**	0.053	0.025	0.043	0.037	-	0.017
**ET**	0.048	0.033	0.032	0.023	0.017	-

**Table 3:** The comparative performance of classifiers in terms of the accuracy used for the classification of IDC(+) and IDC(-) images using standalone descriptors.

MODEL	SIFT	SURF	ORB	Haralick & GLCM
**XGB**	0.858	0.841	0.859	0.850
**MLP**	0.866	0.854	0.844	0.888
**CB**	0.87	0.860	0.862	0.873
**RF**	0.830	0.835	0.830	0.875
**AB**	0.831	0.831	0.843	0.850
**ET**	0.820	0.822	0.828	0.871

**Table 4:** The comparative performance of classifiers in terms of the accuracy used for the classification of IDC(+) and IDC(-) images using the simple fusion of standalone descriptors. Simple fusion refers to combinations, like SIFT-SURF, SIFT-ORB, ORB-SURF and ORB-SURF-SIFT.

MODEL	SIFT & SURF	SIFT & ORB	ORB & SURF	ORB, SURF & SIFT
**XGB**	0.851	0.842	0.846	0.850
**MLP**	0.847	0.851	0.838	0.842
**CB**	0.879	0.862	0.859	0.840
**RF**	0.813	0.826	0.821	0.829
**AB**	0.841	0.837	0.814	0.822
**ET**	0.815	0.802	0.801	0.810

**Table 5:** Comparison with state-of-the-art methods on CXR images. In this table, ACC, P, R, F1-S and AUC indicate the accuracy, precision, recall, F1-score and area under ROC curve, respectively. Bold face scores indicate the best performance while ‘-’ indicates that the specific value is not available. ACC with ‘*’ represents balanced accuracy.

Work Ref.	Method	ACC	P	R	F1-S
Rahman et al. [18]	CNN	0.890	-	-	0.893
Romano and Hernandez [19]	CNN	0.854 *	0.851 *	0.854 *	0.852
CruzRoa et al. [20]	CNN	0.842 *	0.766	0.796	0.718
Wang et al. [21]	CNN	0.890	0.923	0.920	0.923
Chapala et al. [24]	ResNet-50	0.910	-	-	-
Alghodhaifi et al. [34]	IDCNet	0.875	0.810	0.935	0.760
**Present Work**	CB	**0.925**	0.934	0.888	0.907

## Data Availability

The data is publicly available.

## References

[1] Feig S.A., Yaffe M.J. (1995). Digital mammography, computer-aided diagnosis, and telemammography. Radiol. Clin. N. Am..

[2] Sung H., Ferlay J., Siegel R.L., Laversanne M., Soerjomataram I., Jemal A., Bray F. (2021). Global cancer statistics 2020: GLOBOCAN estimates of incidence and mortality worldwide for 36 cancers in 185 countries. CA Cancer J. Clin..

[3] Lowe D.G. (2004). Distinctive Image Features from Scale-Invariant Keypoints. Int. J. Comput. Vis..

[4] Bay H., Tuytelaars T., Van Gool L. SURF: Speeded up robust features. Proceedings of the 9th European Conference on Computer Vision.

[5] Rublee E., Rabaud V., Konolige K., Bradski G. ORB: An efficient alternative to SIFT or SURF. Proceedings of the 2011 International Conference on Computer Vision.

[6] Haralick R.M., Shanmugam K., Dinstein I. (1973). Textural Features for Image Classification. IEEE Trans. Syst. Man Cybern..

[7] Breiman L. (2001). Random Forests. Mach. Learn..

[8] Prokhorenkova L., Gusev G., Vorobev A., Dorogush A.V., Gulin A. (2018). CatBoost: Unbiased Boosting with Categorical Features. Proceedings of the 32nd International Conference on Neural Information Processing Systems.

[9] Freund Y., Schapire R.E. (1999). A Short Introduction to Boosting. Proceedings of the Sixteenth International Joint Conference on Artificial Intelligence.

[10] Doyle S., Agner S., Madabhushi A., Feldman M., Tomaszewski J. Automated grading of breast cancer histopathology using spectral clusteringwith textural and architectural image features. Proceedings of the 2008 5th IEEE International Symposium on Biomedical Imaging: From Nano to Macro.

[11] Dundar M.M., Badve S., Bilgin G., Raykar V., Jain R., Sertel O., Gurcan M.N. (2011). Computerized classification of intraductal breast lesions using histopathological images. IEEE Trans. Biomed. Eng..

[12] Niwas S.I., Palanisamy P., Zhang W., Isa N.A.M., Chibbar R. Log-gabor wavelets based breast carcinoma classification using least square support vector machine. Proceedings of the 2011 IEEE International Conference on Imaging Systems and Techniques.

[13] Kral P., Lenc L. LBP features for breast cancer detection. Proceedings of the 2016 IEEE International Conference on Image Processing (ICIP).

[14] Yasiran S.S., Salleh S., Mahmud R. (2016). Haralick texture and invariant moments features for breast cancer classification. AIP Conf. Proc..

[15] Narayanan B.N., Krishnaraja V., Ali R. Convolutional Neural Network for Classification of Histopathology Images for Breast Cancer Detection. Proceedings of the 2019 IEEE National Aerospace and Electronics Conference (NAECON).

[16] Debelee T.G., Amirian M., Ibenthal A., Palm G., Schwenker F. (2017). Classification of mammograms using convolutional neural network based feature extraction. Proceedings of the International Conference on Information and Communication Technology for Develoment for Africa.

[17] Debelee T.G., Gebreselasie A., Schwenker F., Amirian M., Yohannes D. (2019). Classification of mammograms using texture and cnn based extracted features. J. Biomim. Biomater. Biomed. Eng..

[18] Rahman M.J.U., Sultan R.I., Mahmud F., Ahsan S.A., Matin A. Automatic System for Detecting Invasive Ductal Carcinoma Using Convolutional Neural Networks. Proceedings of the TENCON 2018—2018 IEEE Region 10 Conference.

[19] Romano A.M., Hernandez A.A. Enhanced Deep Learning Approach for Predicting Invasive Ductal Carcinoma from Histopathology Images. Proceedings of the 2019 2nd International Conference on Artificial Intelligence and Big Data (ICAIBD).

[20] Cruz-Roa A., Basavanhally A., González F., Gilmore H., Feldman M., Ganesan S., Shih N., Tomaszewski J., Madabhushi A., Gurcan M.N., Madabhushi A. (2014). Automatic detection of invasive ductal carcinoma in whole slide images with convolutional neural networks. Medical Imaging 2014: Digital Pathology.

[21] Wang J.L., Ibrahim A.K., Zhuang H., Ali A.M., Li A.Y., Wu A. A Study on Automatic Detection of IDC Breast Cancer with Convolutional Neural Networks. Proceedings of the 2018 International Conference on Computational Science and Computational Intelligence (CSCI).

[22] Sanyal R., Jethanandani M., Sarkar R. (2020). DAN: Breast Cancer Classification from High-Resolution Histology Images Using Deep Attention Network. Advances in Intelligent Systems and Computing.

[23] Sanyal R., Kar D., Sarkar R. (2021). Carcinoma type classification from high-resolution breast microscopy images using a hybrid ensemble of deep convolutional features and gradient boosting trees classifiers. IEEE/ACM Trans. Comput. Biol. Bioinform..

[24] Chapala H., Sujatha B. ResNet: Detection of Invasive Ductal Carcinoma in Breast Histopathology Images Using Deep Learning. Proceedings of the 2020 International Conference on Electronics and Sustainable Communication Systems (ICESC).

[25] Debelee T.G., Schwenker F., Ibenthal A., Yohannes D. (2020). Survey of deep learning in breast cancer image analysis. Evol. Syst..

[26] Schwenker F., Roli F., Kittler J. (2015). Multiple Classifier Systems. Proceedings of the 12th International Workshop.

[27] Schwenker F., Dietrich C., Thiel C., Palm G. (2005). Learning of decision fusion mappings for pattern recognition. Int. J. Artif. Intell. Mach. Learn. (AIML).

[28] Bellmann P., Thiam P., Schwenker F., Pedrycz W., Chen S.M. (2018). Multi-classifier-Systems: Architectures, Algorithms and Applications. Computational Intelligence for Pattern Recognition.

[29] Kächele M., Thiam P., Palm G., Schwenker F., Schels M. Ensemble methods for continuous affect recognition: Multi-modality, temporality, and challenges. Proceedings of the 5th International Workshop on Audio/Visual Emotion Challenge.

[30] Boongoen T., Iam-On N. (2018). Cluster ensembles: A survey of approaches with recent extensions and applications. Comput. Sci. Rev..

[31] Faußer S., Schwenker F. (2015). Neural network ensembles in reinforcement learning. Neural Process. Lett..

[32] Geurts P., Ernst D., Wehenkel L. (2006). Extremely randomized trees. Mach. Learn..

[33] Chen T., Guestrin C. XGBoost: A Scalable Tree Boosting System. Proceedings of the 22nd ACM SIGKDD International Conference on Knowledge Discovery and Data Mining.

[34] Alghodhaifi H., Alghodhaifi A., Alghodhaifi M. Predicting Invasive Ductal Carcinoma in breast histology images using Convolutional Neural Network. Proceedings of the 2019 IEEE National Aerospace and Electronics Conference (NAECON).

